# Disinvestment policy and the public funding of assisted reproductive technologies: outcomes of deliberative engagements with three key stakeholder groups

**DOI:** 10.1186/1472-6963-14-204

**Published:** 2014-05-05

**Authors:** Katherine Hodgetts, Janet E Hiller, Jackie M Street, Drew Carter, Annette J Braunack-Mayer, Amber M Watt, John R Moss, Adam G Elshaug

**Affiliations:** 1 School of Population Health, The University of Adelaide, North Terrace, Adelaide, SA 5006, Australia; 2 School of Health Sciences, Faculty of Health, Arts and Design, Swinburne University of Technology, PO Box 218, Hawthorn, VIC, Australia; 3 Menzies Centre for Health Policy, Sydney School of Public Health, Sydney Medical School, The University of Sydney, Coppleson Building D02, Sydney, NSW 2006, Australia

**Keywords:** Australia, Evidence-based health policy, Deliberative methods, Discourse analysis, Disinvestment, Assisted reproductive technology

## Abstract

**Background:**

Measures to improve the quality and sustainability of healthcare practice and provision have become a policy concern. In addition, the involvement of stakeholders in health policy decision-making has been advocated, as complex questions arise around the structure of funding arrangements in a context of limited resources. Using a case study of assisted reproductive technologies (ART), deliberative engagements with a range of stakeholder groups were held on the topic of how best to structure the distribution of Australian public funding in this domain.

**Methods:**

Deliberative engagements were carried out with groups of ART consumers, clinicians and community members. The forums were informed by a systematic review of ART treatment safety and effectiveness (focusing, in particular, on maternal age and number of treatment cycles), as well as by international policy comparisons, and ethical and cost analyses. Forum discussions were transcribed and subject to thematic analysis.

**Results:**

Each forum demonstrated stakeholders’ capacity to understand concepts of choice under resource scarcity and disinvestment, and to countenance options for ART funding not always aligned with their interests. Deliberations in each engagement identified concerns around ‘equity’ and ‘patient responsibility’, culminating in a broad preference for (potential) ART subsidy restrictions to be based upon individual factors rather than maternal age or number of treatment cycles. Community participants were open to restrictions based upon measures of body mass index (BMI) and smoking status, while consumers and clinicians saw support to improve these factors as part of an ART treatment program, as distinct from a funding criterion. All groups advocated continued patient co-payments, with measures in place to provide treatment access to those unable to pay (namely, equity of access).

**Conclusions:**

Deliberations yielded qualitative, socially-negotiated evidence required to inform ethical, accountable policy decisions in the specific area of ART and health care more broadly. Notably, reductionist, deterministic characterizations of stakeholder ‘self-interest’ proved unfounded as each group sought to prioritise universal values (in particular, ‘equity’ and ‘responsibility’) over specific, within-group concerns. Our results - from an emotive case study in ART - highlight that evidence-informed disinvestment decision-making is feasible, and potentially less controversial than often presumed.

## Background

Internationally, measures to improve the quality and sustainability of healthcare practice and provision have become a key concern of policy [1]. As costs and service demands increase against a background of limited resources, priority setting has become a complex and increasingly central component of healthcare policymaking. Within this context, decisions must inevitably be made as to the public reimbursement of new treatments and technologies and the restriction of funding for established services [2].

An approach to the latter concern, ‘disinvestment’ has emerged as a means of improving healthcare outcomes by evaluating existing services that do not provide sufficiently safe, effective or cost-effective care, and re-directing funding to services deemed superior against these criteria [3]. While disinvestment has been the focus of some interest in nations increasingly oriented to healthcare system sustainability, it has been operationalised only in minimal terms in a limited number of jurisdictions [4,5]. Complexities inherent in the identification of candidate technologies and the specific application of disinvestment criteria have curtailed efforts to establish clear procedures that may engender change *and* withstand political challenge. More broadly, the inherently ethical nature of disinvestment decision-making has raised questions as to the appropriate inputs required to support such processes [6,7]. Increasing agreement that stakeholder input is required for disinvestment processes to be representative and accountable has also complicated the establishment of pragmatic mechanisms for disinvestment [8,9].

### Notes on the Australian policy context

At the national (Medicare) level in Australia, a review process of *existing* health care services funded via Medicare, has been established under the responsibility of the Department of Health, including the Medical Services Advisory Committee (MSAC), which makes recommendations to the Health Minister about what medical services offer sufficient safety and (cost)effectiveness to warrant public subsidy. Since 2009, a ‘Quality Framework for Australia’s Medicare Benefits Schedule’ has been developed whereby individual health care practices face review as to their appropriateness for continued public subsidy at current levels. Potential outcomes from a review include: an amendment to the item description such that it better captures the patient group/s most likely to benefit from any procedure; an increase, decrease or maintenance of the fee; or a complete stop to public funding of the item [3,10]. The present study is aligned within this general policy context.

With these concerns in mind, the present study develops and tests a model whereby different forms of evidence are incorporated into a disinvestment policy process. More specifically, we sought to operationalise a potential disinvestment policy model incorporating evidence of safety and effectiveness, a comparison of policies from a range of jurisdictions, ethical and cost analyses and the informed deliberations of stakeholders with regard to a specific case-study technology: assisted reproductive technologies (ART) with a particular focus on differential effectiveness by age.

### ART – a case study

Australians utilise a high number of ART cycles per million population when compared to other countries – partly the result of essentially unlimited public subsidy for ART services (a contrast to many other similarly developed countries). Australians are eligible for this subsidy regardless of their age or any prior treatment attempts. However, as ART in Australia are offered primarily through the private sector, there remains a patient-borne ‘gap’ payment beyond what Medicare (the nation’s universal health insurance scheme) will subsidise. Once a patient’s out-of-pocket expenses reach a threshold amount in any given year, any further gap payments are covered by government through a Safety Net program. The combination of these factors has led to significant cost escalations for Medicare (tax-payers). Hence, the public subsidy of ART has been a perennially contentious health policy issue in Australia. The Australian government – regardless of the party in power at the time – has periodically entered into policy debates around access criteria for ART services (a situation mirrored in the international experience) for more detail see [11,12].

Assisted reproductive technologies (with a particular focus on the impact of maternal age and number of cycles on treatment cost and effectiveness) were selected as a potentially useful case study on which to trial an engaged disinvestment process – for two reasons. First, ART meet multiple criteria on a proposed framework for identifying disinvestment candidates [9]: there exists substantial temporal and geographic variation in ART treatment provision, in addition to evidence of differential effectiveness across patient sub-groups [11]. Second, as introduced above, considerable public debate and lobby group opinion in relation to previous government-mooted subsidy restrictions in Australia [11,12] suggest that ART represents fruitful ground for analysis of the social and ethical evidence needed to inform a representative disinvestment decision.

In light of these reasons, and to incorporate the different forms of evidence flagged by them, a dual approach to data generation was adopted in this study. First, a systematic review assessed evidence of differential ART safety and effectiveness according to maternal age, paternal age and number of treatment cycles [11]. This review was supplemented by an analysis of ethical considerations relevant to a potential disinvestment decision based on ART effectiveness and maternal age [13] and the incorporation of a recent Australian analysis of ART cost-effectiveness by maternal age and number of treatment cycles [14]. In turn, this evidence base informed deliberative forums attended by vested stakeholders (ART clinicians and consumers) as well as by non-partisan citizens. Guided by the ideals of deliberative democracy, these forums involved the provision of detailed information to support participants’ deliberation on the question of how best to structure the public funding of ART, and were designed to elicit colloquial and experiential knowledge to enhance the structured evidence base arising from the review and supplementary analyses.

Deliberative processes are underpinned by the theory that an appropriately informed sample of the population can deliberate productively with a view to offering contributions to policy development that are reflective of broader values. Used productively in other contexts to canvass community and stakeholder perspectives on health-related priority setting [15-17], deliberative methods are held to both improve and legitimate policy directives [18]. In the context of disinvestment decision-making where, it may be argued, there is a particular imperative for “health services to be accountable to users as taxpayers, voters and consumers” [19], deliberative methods represent a more transparent means of addressing the complex, ethical nature of resource allocation and policymaking.

In this paper we outline the findings of stakeholder engagements that sought to incorporate technical, experiential and colloquial evidence within a hypothetical disinvestment context. In doing so, we propose a disinvestment model that stands to enhance more conventional evidence-based policy with informed stakeholder engagement to support ethical, democratic health governance.

## Methods

Below is a summary of the methods employed in this study. A detailed account of the research process is published elsewhere [6].

### Evidence generation

The first phase of the project entailed conducting a systematic review of ART [11]. This review utilised contemporary health technology assessment methods and policy processes, as appropriate for the Australian context. The protocol was assessed by content experts, and focussed on evidence of effectiveness associated with maternal age and number of treatment cycles. Key outcomes of the systematic review included findings that ART effectiveness decreases with advancing maternal age (noting a particular decrease after age 42) and that, across all age groups, ART effectiveness decreases slightly with each successive cycle of treatment. These findings were consistent with the economic analysis consulted [14], which found that the cost-effectiveness of ART decreases with both treatment cycle number and, in particular, female age. An ethical analysis was conducted to canvass further relevant considerations. It described different possible conceptions of medical need as well as different respects in which these frameworks, as well as treatment effectiveness and maternal age, could be considered relevant to ART funding policy [13,20].

### Deliberative engagements

In the second phase, the evidence generated was presented at a series of deliberative stakeholder engagements held in Adelaide, South Australia with groups of ART clinicians, non-partisan citizens and ART consumers (past patients). Each stakeholder group attended two engagement sessions (two ‘rounds’ of engagements spaced a number of weeks apart) in which they were asked to deliberate on the evidence presented, and to generate (and justify) a preferred approach to the public subsidy of ART in Australia^1^. These engagements incorporated key elements of deliberative methods, including the provision of accessible information by content experts, facilitation by an independent professional, small and larger group deliberation and the generation of policy guidance [21].

An outline of the evidence described above was presented at each engagement by the research team, who then remained in attendance to clarify technical and factual issues as they arose. Participants were also presented with an outline of the approach to ART funding currently operating in a range of other national contexts including Israel, Canada, Denmark and the UK. The funding structure applied in New Zealand was also presented, generating discussion around that country’s use of subsidy criteria which include a potential patient’s age, body mass index (BMI) and smoking status [22].

In light of the information presented, participants were asked to consider the question: *Should the criteria for public funding of ART be changed? If yes, why? If no, why not?* Each group was also encouraged to address broader issues around the barriers and facilitators to disinvestment and the (potential) role of stakeholders within both ART and health policy decision-making, more broadly.

In the second round, outputs from all first round engagements (i.e. the perspectives of each stakeholder group) were reported to all participants and allowed to inform their subsequent deliberations.

The decision to separate stakeholder groups supported participants to voice partisan opinions freely, without fear of the impact of different degrees of expertise [23]. At the same time, the ‘feeding back’ of other groups’ deliberative outputs brought together a range of (potentially conflicting) perspectives [24], which were ultimately presented to relevant policy advisors for consideration.

### Recruitment

A detailed account of the recruitment process is outlined elsewhere [6]. Participants in the consumer forums were purposively recruited on the basis of relevant experience; 9 participants attended Round 1 and 7 returned in Round 2. Community forum participants were randomly sampled (stratification criteria included gender, age and household income) and 14 attended Round 1 while 10 returned for Round 2. Clinician participants were purposively recruited on the grounds of relevant technical experience and as nominees from relevant medical bodies; 8 attended Round 1 while 6 returned for Round 2.

While the sample size for the community engagements was within the range deemed ideal for citizens’ juries [25,26], the consumer and clinician forums were slightly smaller owing to the specificity of recruitment requirements (including selection criteria designed to minimise potential harm to past patients). Nonetheless, the sample sizes allowed for fulsome deliberation around a variety of inputs and opinions, and for active participation in “communicative processes and will formation” [27].

The project received ethics approval from the University of Adelaide’s Human Research Ethics Committee. Participants were assured that their contributions to deliberations would remain anonymous, and that they were at liberty to withdraw their participation at any time. Consent for an experienced stenographer to transcribe deliberations was obtained from all in attendance, and an honorarium was paid to participants at the conclusion of each forum.

### Approach to data analysis

Transcripts of the deliberative forums were analysed thematically [28,29]. Given the project’s dual focus on the specific case of ART, and broader questions around approaches to disinvestment, analysis attended to both the deliberative and analytical outputs of the engagements [30]. That is, we aimed to represent the content of each deliberation, while engaging with the broader policy implications of the perspectives articulated.

Analysis of each group’s deliberations is presented separately to illustrate areas of within-group disagreement and consensus. To highlight pervasive themes, we attend to participants’ responses to questions concerning (1) the key values that should inform potential ART subsidy restrictions, and (2) whether it would be appropriate to restrict subsidies on the basis of personal patient characteristics (including BMI, smoking status, maternal age, or number of treatment cycles). Participants’ perspectives on patient contributions to the cost of ART treatments are also addressed and insights applicable to broader disinvestment questions are considered.

## Results

### Consumers

#### Values underpinning arguments for and against subsidy restrictions

It might have been expected that consumers of ART – particularly those whose treatments resulted in successful live births – would be ‘protective’ of funding in this area, and reluctant to see restrictions applied in a domain with such personal resonance [31]. However, deliberations in the consumer forum were characterised by strong agreement that health resources are limited, and that some subsidy restrictions may therefore be justified in the area of ART, particularly when advancing maternal age significantly reduces the likelihood of positive treatment outcomes. Participants across the group recognised the finite nature of health resources (“We’re not a bottomless pit”– Linda) and demonstrated a nuanced understanding of the notions of both ‘opportunity cost’ (“I think you can’t fund everything, you have to have criteria” – Kate) and ‘capacity to benefit’ (“I wouldn’t expect the government to give me three fully funded [ART treatment] cycles with my chances so small” – Kylie). There was agreement across the board that these concepts were highly relevant to public subsidy decisions.

Questions around the status of ART as a “want or a need” also informed – and polarised – the subsidy debate. On the one hand, the depiction of ART as a non-essential ‘choice’ supported calls for subsidy reductions and increased patient contributions to treatment costs.

LARA: You just save, save, save to accomplish what you want because … it’s the difference between a want and a need.

RITA: I don’t think the government should entirely fund all of it because … we’ve all got a medical condition, that’s why we’re into IVF, but it’s more a case of personal wanting … It’s not that you have a heart condition and you need that to live.

On the other hand, the notion that people do not ‘choose’ infertility repositioned ART as a medical ‘need’ that should be funded like any other.

DEANNE: [Subsidy restrictions are] completely horrifying because I didn’t choose my situation.

DAWN: If somebody has a mental health condition, do we only support them to get better if they meet certain criteria, or do we say this person has a need … we need to match the health provision with the medical need?

Despite this debate, there was broad agreement that any kind of restriction to ART subsidies should be based upon medical evidence. Other grounds for restrictions, in the absence of scientific ‘facts’, were pervasively denounced as ‘arbitrary’ and therefore insupportable.

PAULINE: You can’t come up with an arbitrary figure and say [age] 43 or 45, it should have evidence behind it.

DEANNE: If it’s not backed up by scientific facts and proof, then I feel really, really resentful that a government is going to make a decision on whether I’m a mother or not. I find that arrogant.

‘Medical evidence’ with regard to treatment effectiveness was depicted as distinct from ‘clinical judgement’. While ART consumers supported the use of clinical effectiveness data in informing subsidy schedules, they were more reserved in their endorsement of ‘medical opinion’ as a basis for funding limitations. While these arguments were primarily proffered on the grounds of “transparency”, there was also concern that a blurring of clinical judgement and doctors’ interests may not always encourage prudent financial, or emotional, investment.

PAULINE: Any sort of restriction should be scientifically based. [A test of ovarian reserve], maybe something like that. I think it puts a restriction on it for funding and it also gives the women a bit more insight and transparency and is not giving them false hope.

#### Restrictions based upon patients’ social/moral characteristics

Participants in this forum suggested that no access to subsidised cycles of ART should be allowed for current drug users or people who have been convicted of child abuse.

Beyond these specific concerns, the ART consumer forum did not support the introduction of subsidy restrictions based upon patients’ social or moral characteristics. While discussion of ‘patient worthiness’ was evident, this theme generally did not appear in accounts arguing for the exclusion of ‘undeserving’ consumers. Only one participant suggested that issues of choice reduce worthiness (“I felt guilty accessing it because my partner had chosen prior to me, having a vasectomy” – Kate), while another argued that already having children (seen as a choice after having ‘met the need’ to be a mother) could be a legitimate grounds for restriction. More frequently, (often autobiographical) accounts of patients who *had* ‘taken responsibility’ served to legitimate continued public expenditure on ART for those who have ‘done their part’ within the reciprocal taxpayer contract.

DEANNE: I have worked hard for this country and worked hard for this State. I’m a little bit pissy I’m paying so much [referring to co-payments]… My BMI was low and I’d never smoked, never drank. I kind of felt like “I have done my part. You do your part”.

#### Restrictions based upon age and treatment cycle number

The consumer forum agreed to outer female age limits for ART treatment subsidies: no access under 21 or over 45. This agreement was reached in the second engagement session after lengthy discussion in the first as to whether age 42 might be an appropriate ceiling (interestingly, at least three participants were approximately this age when they received ART treatment).

Ultimately, the forum reached its strongest agreement around the notion that ovarian reserve (in conjunction with other physiological markers of likely treatment effectiveness) is the most appropriate basis for limiting subsidy, and preferable to limits based upon age or cycle number. This agreement was underpinned by the understanding that such policy decisions should be both ‘individualised’ and ‘grounded in medical evidence’.

JOHN: I think you have to assess case by case and say “Look, what [are] the health issues, what is the ability of the person to have a baby”.

While essentially representing a ‘capacity to benefit’ argument (“the ability of a person to have a baby”), such accounts called for more nuanced restrictions than blunter, age-based limitations. However, although these arguments appeared to place considerable decision-making responsibility in the hands of clinicians, participants were keen to hold doctors’ powers in check.

DEANNE: There has to be some … medical ethics involved here [because] they’re benefiting from you being a repeat client.

Although most participants’ arguments in favour of restrictions emphasised the capacity to benefit and issues of financial prudence (the notion that it is a ‘bad investment’ when a patient’s likelihood of success is low), one participant suggested that age limits could have additional benefits with regard to patients’ psychological ‘closure’.

DEANNE: I think people need to have some closure point, I think that is psychologically important, and that it would be better put to bed if there’s scientific fact based on that closure point rather than a government saying “This is your closure point because we are the government of the day”.

#### Restrictions based upon BMI/smoking status

Discussion of the funding model operating in New Zealand at the time the forums were held focused on the legitimacy of limiting ART subsidies on the grounds of BMI and smoking status.

The recurring theme of ‘taking responsibility’ informed arguments in the consumer forum in favour of limiting ART subsidies for patients whose BMI and smoking status pose risks to the success of their treatment. Once again, a reciprocal construction of health expenditure was evident:

DAWN: [The funders] could be saying “We’ve done our part, what have you done? You’re smoking, you’re overweight, your BMI is through the roof. You are a huge risk to us as a success”

DEANNE: If it’s going to be publicly funded I think the client has a responsibility to play their part in the success of the fertility treatment.

More commonly, however, accounts advocating limits based upon smoking status and BMI oriented to the likelihood these factors will reduce treatment success, or pose risks to the unborn child.

KATE: [It shouldn’t be about] discriminating because you are a smoker or obese, but that your chances of getting pregnant because you are obese or a smoker are much less than if you were not.

RITA: There [are] statistics to say you are affecting your baby. You may not, but the statistics say that your baby can be affected.

Although limitations based upon BMI and smoking status were broadly accepted, there was concern that such restrictions might unfairly discriminate against those facing issues ‘beyond their control’. The dominant concern that patients ‘take responsibility’ was circumvented in accounts of circumstances in which BMI or smoking were deemed beyond a patient’s full responsibility or power to change.

KYLIE: I think you have to be careful that it’s not seen as a form of discrimination … there are conditions that cause them to be overweight not by their own actions or choice.

A more explicit depiction of obesity and smoking as ‘medical problems’ supported arguments against such restrictions, allowing the invocation of ‘slippery slope’ arguments (“Ethically, I mean do we stop operating on people who are overweight for other things?” – Kylie) and eventual agreement that weight control and smoking cessation support should become *part* of ART treatment programs, rather than serve as criteria for entry into them.

#### The issue of co-payment

The consumer forum achieved a consensus opinion that patients should (continue to) contribute a co-payment for their ART treatment. This position was informed by the pervasive emphasis on ‘taking responsibility’, and linked to the (minority, but repeated) opinion that ART represents a ‘choice’.

KATE: We weren’t rich, we worked hard. It’s your choice.

A pervasive concern that treatment options should be equitable led many in the forum to advocate a means test for co-payments (namely, that a patient’s contribution should be relative to their capacity to pay).

PAULINE: We’d be setting out babies only for the rich.

Eventually, broad agreement was reached that a prohibitive blanket cost is unjust and unsupportable if it puts ART beyond the reach of many.

KYLIE: It’s unfair just because you are not in a good job or a white collar job that you’re punished, that you can’t access these services.

### Community

#### Values underpinning arguments for and against subsidy restrictions

While the consumer forum emphasised notions of patient ‘responsibility’, the community forum was characterised by the perspective that the health care system must ensure the needs of *all* patients are met. Only one participant questioned the appropriateness of subsidising ART per se (“A child is a gift … [infertility is] the numbers you draw sometimes” – Roger); beyond this there was broad agreement that infertility represents a need that should be funded “like any other medical condition” (Ron).

Participants were particularly concerned that subsidy restrictions may be gender discriminatory given that contradictory workplace and fertility imperatives put women in “an impossible situation” (Martha) with regard to the timing of conception and childrearing. There was also general agreement that all infertile patients should be able to access ART treatments, and that wealth should not be a barrier in this regard:

MARTHA: You are either infertile or not. Whether they are rich infertile or poor infertile I think they should be on the same level.

#### Restrictions based upon patients’ social/moral characteristics

Participants unanimously agreed that no access to subsidised ART treatments should be allowed for convicted paedophiles, and all but one participant agreed that convicted child abusers should be similarly excluded. It was agreed that users of illicit drugs should be restricted, but allowed to appeal their restriction on the basis of mitigating circumstances (e.g. number of years clean).

Beyond these issues, two divergent opinions emerged (and were not reconciled) in the community forum around whether it would be appropriate to restrict ART subsidies with regard to patients’ social and moral characteristics. One group of participants indicated a willingness to “screen” ART recipients to ensure that their treatment is being sought “for the right reasons” (Esther).

CURTIS: Some parents aren’t necessarily having children for wholesome reasons and [maybe] there can be some sort of screening around that, particularly if the baby bonus outweighs the costs of the IVF.

One participant introduced the notion that characteristics of the children resulting from ART may (or may not) justify the costs of their conception.

DOUG: It doesn’t work because what you get at the end, assuming this person goes to work which is no guarantee, there’s no guarantee that person will go to work and pay taxes … so you have these resources going in and no guaranteed income at the end of it.

Another group of participants voiced strong rebuttal to these perspectives, arguing the abhorrence of policy grounded in subjective valuations of prospective parents or children.

SARAH: Who in their right mind could price a child or anybody’s life? … [We] do not get to decide who is more valuable, who is not. We all eat, we all go to the toilet, we all breathe, we all contribute in some way to society. How do we get to decide that? Why should governments play God?

#### Restrictions based upon age and cycle number

Participants in the community forum agreed upon an upper age limit of 45 years and a lower age limit of 18 years for subsidised ART treatment. More restrictive limits on the basis of maternal age and number of cycles generated considerable debate, a pervasive perspective being that treatment decisions should be “individualised” rather than being based upon population statistics.

RON: I think it’s a very individual issue about whether the treatment can be deemed to be appropriate for the patient, not based on statistics.

After much deliberation, and confirmation from the researchers in attendance that most patients do not attempt more than seven treatment cycles, a majority of participants agreed that patients should be able to access a maximum of ten subsidised cycles. This framework was subsequently refined, and broad agreement emerged that an appropriate structure would allow five subsidised cycles *per live birth* up to a maximum of two children born of IVF. Within this model, a majority of participants supported a limit of two treatment cycles for patients over the age of 40, given that after this point the likelihood of treatment success “drops off so much” (Curtis).

The notion of outcome probabilities also informed questions (ultimately unresolved) around potential exceptions to the proposed limit of two subsidised cycles after the age of 40. A question arose as to whether it might be more appropriate to subsidise more than two cycles for women over 40 who had *already* had a child on the grounds that selection bias suggests they are “a more reliable bet”. Conversely, a question also arose as to whether extra cycles for women over 40 should be limited to those who had not yet had success, on the grounds that they faced greater need.

#### Restrictions based upon BMI/smoking status

Community forum members demonstrated more openness to subsidy restrictions based upon BMI and smoking status than either consumers or clinicians. Concern around ‘singling out’ ART for restriction on these grounds was a minority opinion in this context.

ANNIE: What about heart disease? We all know we should exercise and not smoke, and yet we still keep funding for [that].

More commonly, willingness to restrict on age and smoking was justified and supported on a variety of grounds. For example, one participant emphasised the general importance of (prospective) mothers ‘taking responsibility’.

MEGAN: What I like about [restricting on these grounds] is the fact there is some input from the mother, she can’t just sit back and expect it all to happen to her, she has to make an effort.

More pervasive justifications rested on the impact of smoking and obesity on the likelihood of treatment success and on the health of the unborn child.

ALLAN: the smoking part of it, we know that makes a pretty dramatic impact on their ability to carry to term, the health of the child, even after the child has been born, passive smoking around the child, I think for me that would be a gateway hurdle.

For one participant, these risks were specifically articulated in economic terms:

CURTIS: We all know that for a smoker it increases the risk of a deformed pregnancy … that it is going to be more public money that’s going to have to go into that and this is a risk we could actually stop now by not allowing the IVF.

The forum agreed that they would support restrictions for current smokers and patients whose BMI indicates they are obese, with the option for patients to “appeal to a board” if their obesity reflects exceptional circumstances. It was noted, however, that an appeals board could, in itself, represent an economic burden (“The board might cost you more than the funding would” – Esther).

#### The issue of co-payment

It was broadly agreed that co-payments for ART treatments should be retained. For some participants, a co-payment was deemed an important means of encouraging earlier, unassisted reproduction in the wider population as opposed to ‘reliance’ on ART.

MARTHA: [T]o encourage people that may have the potential for fertility as they are younger but not as they get older, I think there should be a co-payment.

For others, co-payments represented a means of proving one’s commitment to having a child and one’s responsibility as an aspiring parent.

MEGAN: If they want it badly enough, they should be making some kind of contribution.

Arguments emphasising dis/encouragement, personal responsibility and the concern with ‘equity’ coloured the debate around how a co-payment schedule should be structured. As deliberation progressed, ‘responsibility’ arguments supporting calls for a co-payment on all cycles gave way to concern that the prohibitive nature of *any* co-payment might reduce equity of access. After canvassing a ‘safety net’ option (the suggestion that patients stop being required to make a co-payment once they have reached a certain level of expenditure), for which there was majority agreement, nine out of ten participants agreed to a participant’s proposal that patients be given two treatment cycles free of charge, then charged a co-payment for subsequent cycles. This proposal was seen to encourage both equity of access *and* patient responsibility. In turn, this payment structure was argued to encourage earlier natural reproduction (to avoid later-cycle co-payments) and to make ART accessible to younger infertile couples who would not have to delay treatment while saving for their co-contribution.

### Clinicians

#### Values underpinning subsidy/coverage restrictions

Clinicians’ deliberations centred on accounts highlighting the significance of ART outcomes (see [32] for a complete account of clinicians’ deliberations around evidence of ART effectiveness). There was strong agreement that ART funding should be approached ‘no differently to the funding of treatment for any other medical condition’, given the comparable benefits.

GEOFF: [Deliberations about the funding of] ART still is based on a concept that it’s not as serious as cancer or Alzheimer’s or whatever.

While only one clinician mobilised the language of ‘rights’ to advocate for ART funding (“The ability to reproduce is a basic human right” – Helen), all participants emphasised the significance of ART outcomes as a more than adequate return on a funding investment. Supported by the underlying value that health funds should not be wasted, clinicians argued the superior utility of ART funding in comparison to other areas of health expenditure.

GEOFF: It’s a very interesting exercise to count the loss of QALYS^2^ if there is insufficient fertility treatment [and] if you take that seriously compared to many parts of medicine, there should be a big increase in funding for fertility service[s].

Concern around the potentially discriminatory implications of ART restrictions also coloured clinicians’ deliberations. Clinicians emphasised a need to protect themselves in this regard (with regard to age limits, doctors “do get cases taken to antidiscrimination all the time” – Jane), and the implications for women more generally (“The 50-60 year old husband of a much younger second wife, and he’s had his vasectomy and he’s now perfectly able to come along and have IVF treatment without anyone arguing about it but his 45 year old ex-wife is not able to” - Helen).

#### Restrictions based upon patients’ social/moral characteristics

Clinicians did not deliberate extensively around this issue. One clinician raised concerns about a 50-year-old parent’s capacity “to run around” after their seven-year-old child, but the majority would not countenance the notion of socially-derived restrictions.

#### Restrictions based upon age and cycle number

Once again, concern around discrimination (specifically framed in terms of unequal access to significant, life-changing technology) underpinned deliberations. Like the consumer and community groups, clinicians emphasised the need for individualised assessment rather than the local application of population statistics.

HELEN: I take every person on face value and individual characteristics. To put these markers on it is dangerous when you talk about individuals.

In general terms, the clinicians rejected calls for blanket age or cycle limits. However, there was agreement that ovarian age would represent a more legitimate basis for restrictions than chronological age, if limitations were deemed necessary.

HELEN: I replace age with fertility ovarian reserve assessment. [It’s] a more inclusive assessment of chance of success rather than just a blanket age.

While deliberations were coloured by broad agreement that the ‘art of medicine’ is paramount in this domain (‘I would hate to have laws dictating what we can and can’t do in this area’), one clinician was in favour of an explicit upper age limit. A desire for an externally imposed justification for not subsidising ‘futile treatment’ underpinned this position:

JANE: I would like Medicare to tell us that [45 is the limit]; not saying no, they can fully fund it themselves.

ARTHUR: Why 45?

JANE: It’s futile treatment.

#### Restrictions based upon BMI and smoking status

Deliberations around the legitimacy of restricting on ‘lifestyle factors’ were particularly heated in the clinicians’ forum.

Some participants argued in favour of women being required to “correct the correctable” (reducing BMI, smoking cessation) before ART. While framed in terms of costs and benefits, these arguments emphasised a need to act in the best interests of the child while also maximising the likelihood of treatment success.

JULIA: The chances of an IVF cycle succeeding in somebody who is currently smoking is 50% compared to a non-smoker. Should they not be responsible to society and the taxpayer and stop smoking for two reasons: to increase their chances of pregnancy for themselves and society and to increase the wellbeing of their unborn child?

For one participant, explicit restrictions in this regard were framed as a means of ‘backing up a doctor’s call’ in cases where clinicians are cognisant of the likely impact of obesity or smoking yet feel unable to refuse treatment on these grounds.

JANE: Part of me would like the idea ‘the computer says no’ and you have to go and lose some weight.

Strong objections were levelled at each of these arguments by other forum members. For several clinicians, concerns with such restrictions were practical: BMI and smoking status were depicted as arbitrary measurements that may bear no relation to potential treatment outcomes.

HELEN: [BMI and smoking] are arbitrary characteristics about human beings… If you are a smoker with an obstructed fallopian tube, why is the smoking causing you not to get pregnant?

For others, a deeper ethical concern around ‘punishing people for their lifestyle’ underpinned their position, which was bolstered by a depiction of obesity and smoking as ‘medical disorders’ only partly within a person’s control.

GEOFF: I [think] very much in principle that people should have exactly the same healthcare. …So far in healthcare we have never prejudiced people for their lifestyle. … BMI is increasingly recognised as a medical disorder which is only partly under control of people. We never apply the principle: “Oh you’re too big, you are not going to have surgery at all”. That is really shaking the foundation of how we provide healthcare.

Fears that restrictions of this kind would unfairly ‘single out’ ART treatments prompted participants to draw comparisons with other areas of healthcare expenditure and to invoke the ‘slippery slope’ of limiting medical subsidies on the basis of lifestyle ‘choices’.

JANE: You can relate that back to the $1.3 billion being spent on statins to lower cholesterol. Will we say “stop eating the high cholesterol food?”. Don’t spend the $1.3 billion because you want to eat prawns?

GEOFF: Basically the neurosurgeon across the street [could] say “We’re not going to treat your brain haemorrhage because you were speeding”. That’s the slippery slope.

While pervasive concern was voiced around the ‘unfairness’ of restricting on these grounds for ART alone (“[That] would be all very well if every other medical intervention had the same criteria applied to it” – Helen), others countered that such restrictions *do* (perhaps quite rightly) apply.

LYDIA: [T]here are some other situations where they do take that into account; for example transplantation and there are other medical conditions, where you may not get your heart transplant.

Debate was intense, and involved a range of competing conceptions of the costs and benefits (medical, social and moral) of restricting treatment subsidies on the basis of ‘lifestyle factors’.

#### The issue of co-payment

All participants agreed that ART patients should continue to be responsible for a financial co-contribution. There was broad consensus that co-payments represent a means of ensuring that patients ‘value’ the service provided.

RANDAL: As soon as it’s completely free, it’s often not valued.

JULIA: It’s abused.

At the same time, the clinicians agreed that access to ART should be ‘equitable’, a value that underpinned calls for a co-payment structure to reflect prospective patients’ capacity to pay. Only one participant argued that a universal co-payment supports *equality* because ‘everyone is treated the same’. All others advocated a co-payment structure whereby contributions are means tested, or linked to one’s financial capacity, so that treatment does not become beyond the reach of many.

RANDAL: It’s equitable, everyone gets a shot.

HELEN: They don’t … They don’t all get a shot.

GEOFF: The top end [do]

LYDIA: Some people do not get the shot.

Concern around the prohibitive nature of co-payments (“There are people who would not dream of it because they don’t have the money” – Geoff) and the lack of opportunity for lower income patients to seek fully funded treatment in a public environment ultimately led the forum to advocate for one free treatment cycle for those with demonstrated need.

HELEN: We are saying the icing on the cake would be to add in the possibility of a cycle for those who really can’t afford it.

To summarise the outcomes discussed in this section, the general position reached by each stakeholder group is represented in the table below.

## Discussion

In combination, the outcomes of these deliberative engagements represent an informative contribution to funding policy debates in this arena. Our results inform the application of this engagement method, (a) in disinvestment deliberations generally, and (b) specifically in terms of ART, including suitability of the method for contested (ethical) domains.

Firstly in relation to general contributions, it was clear that all participants understood the finite nature of health funding, and the notion that difficult decisions must be made when treatment costs expand within a context of limited resources. This underscores a key tenet of deliberative democracy: that all citizens, when provided with sufficiently detailed background material, can participate thoughtfully and meaningfully in the process of policy formation [27].

More significantly, it was evident that participants were open to genuine consideration of funding options that did not always align with their own interests. The willingness of ART consumers and clinicians to countenance restrictions, and for community members cognizant of ‘opportunity costs’ to be open to continued expenditure, speaks to the capacity of invested stakeholders in taking up the challenge of deliberative democracy [33]. Reductionist, deterministic characterizations of stakeholder ‘self-interest’ proved unfounded as each group sought to prioritise universal values (in particular, ‘equity’ and ‘responsibility’) over specific, within-group concerns. This result has significance, as previously it has been documented that decision-makers reveal an inclination to shy away from disinvestment decisions due to their allotting weight to the voices of resistant, vocal minorities [12]. Our results suggest such weighting to be disproportionate to the broader stakeholder response and incongruent with an aversion to act based on perceived risk of stakeholder backlash.

With ART forming the background context, numerous outcomes were necessarily ART specific. Yet agreement on a range of broad values was evident across the engagements, and these have implications for the transferability and applicability of this method beyond ART. As we have shown, each forum emphasized a need for subsidy restrictions to be ‘grounded in medical evidence’ rather than governmental decree. Interestingly, *population-based* medical evidence of a drop in treatment effectiveness with advancing maternal age (suggesting the potential usefulness of an age-based subsidy restriction) was somewhat undermined by another shared value: the importance of *individualizing* treatment decisions. A pervasive feature of contemporary health discourse [34], this latter principle underscored the preference of the consumer and clinicians’ forums that any potential restriction to be based upon (individual) physiological markers rather than maternal age or cycle number. Future research could elucidate the practicality of this suggested approach by outlining the state-of-the-science behind tests for ovarian reserve and other physiological markers, and asking, for instance, whether test accuracy is superior to age in predicting ART prognosis.

On the controversial topic of whether BMI and smoking status represent legitimate grounds for subsidy restrictions, not one forum reached consensus. In each group, concerns around fairness (‘singling out’ ART; punishing people ‘for their lifestyle’) came up against concerns around ‘maximising treatment effectiveness’ and ‘the best interests of the child’. These principles are applicable within ART and beyond. The fact that the deliberations of each group were so similar underscores the capacity of ‘lay’ people to contribute to the ethical aspects of health policymaking [35]. Despite disparate levels of technical knowledge, the groups were similarly equipped to engage with the social and moral complexities of restricting on these grounds.

The importance of canvassing different groups of stakeholders separately [23] also became evident when differences across the groups were observed. For example, while the clinicians agreed that infertility should be treated ‘no differently than cancer’, both the consumer and community groups engaged in heated debate on this issue – debate that may have been less freely expressed in the presence of medical experts. Similarly, while the community and clinician groups argued that the ‘judgment of doctors’ should be paramount in decisions regarding likely treatment effectiveness, consumers were less deferential, emphasizing a need for guidelines to ensure that doctors are ‘transparent’ about costs and likely outcomes. Again, such resistance to wholesale ‘doctor deference’ may have been expressed less explicitly in the presence of clinicians.

Ultimately, each stakeholder forum reached a different decision as to their preferred ART funding structure, emphasizing different guiding principles (See Table 1). For the consumer group, an outer limit of 45 years with means-tested co-payments represented a model informed by concern with the financial aspects of the ART journey, and a unique framing of an ART age cut-off as important for psychological ‘closure’. For community members, a limit to the subsidized funding of 5 cycles per child (up to a maximum of two children), restrictions on smoking and BMI (with room for appeal) and two free cycles with co-payments required thereafter, arose within an often genuinely hypothetical deliberation process that attended to ethical issues around equity of access, opportunity cost and capacity to benefit. The clinicians’ model, advocating the continuation of co-payments with the addition of a free cycle for those unable to pay, also oriented to equity of access, their desire for a mandated age ceiling (45) argued to support them in ‘making their call’(i.e. to deny ART treatment on grounds of medical futility).

**Table 1:** The ART public funding arrangements commended by different stakeholders

	**Age limit**	**Cycle limit**	**Other restrictions**	**Payment structure**
**Consumers**	No publically subsidized access <21 or >45	-	-	Means-tested co-payment for all cycles
**Community members**	No publically subsidized access <18 or >45 and; limit of 2 cycles >40	5 cycles per child to max. 2 children	Current smokers; BMI in obese range (option to appeal); Convicted paedophiles, child abusers; Illicit drug users	2 free cycles; co-payment on subsequent cycles
**Clinicians**	No publically subsidized access >45	-	-	Co-payment for all cycles; option of free cycles for those unable to pay

As these outcomes illustrate, all stakeholder groups advocated in favour of patient co-payments for ART treatment programs, which may reflect the broader experience Australians have with co-payments across the health care system. This compares to the findings of Rauprich [31] whose survey canvassed patient and professional opinions on the financing of ART in Germany. In that study, co-payments for ART were deemed acceptable by only one-third of patients, two-thirds of ART physicians and three-quarters of other groups (including academics, social lawyers and health politicians). Among these groups, a preference for full public subsidy was strongly correlated with the conceptualization of infertility as ‘a disease’ or ‘medical need’. Our findings contrast with those of Rauprich et al., in that our patient and clinician participants, who most frequently framed infertility as an issue of ‘medical need’, were as supportive of continued co-payments as members of the broader community.

More noteworthy is the finding that each forum explicitly incorporated into their preferred funding arrangement policy elements aimed at increasing equity of access. Again, although this principle was articulated specifically for ART it may well be a broader concern. While the policy elements differed from group to group, this finding echoes concerns noted elsewhere [31,36] that co-payments currently do, but ought not to, put ART out of the reach of the less affluent. This consensus is indicative of a concern not currently addressed in the Australian context, namely that access to fully subsidised ART treatment within the public system is severely limited and co-contributions for private patients are not differentiated by income.

In terms of the full range of opinions regarding ART subsidies, our study was limited to the extent that all participants in the consumer forum had been successful in their ART journey; for ethical reasons we did not recruit unsuccessful former patients. In addition, the study was informed by ethical and cost analyses, as well as studies of treatment effectiveness, but did not draw media representations of ART (arguably an important informant of public discourse on this topic) into the discussion (for analysis on this element see Street et al [12]). However, by involving a range of stakeholders including ‘average taxpayers’, a group rarely canvassed within health policy development [26], this study indicates important possibilities for ensuring accountability in the health policymaking process – a particularly important concern in the context of what are largely ethical decisions.

Selecting ART as a case study was strategic given the ethically charged nature of the debate in Australia and elsewhere. The engagement sessions proved highly instructive, with outcomes being relevant for ART specifically, but also in capturing underlying principles that have broader relevance in disinvestment decision-making. It has been argued that, for disinvestment decisions, a different level of evidence is required than that required for investment, in large part because of the social and/or political baggage involved in ‘taking something away’ [7,10,37]. Indeed, the nature of such decision-making may also require different *kinds* of evidence than those typically incorporated, namely the qualitative, experiential, socially-negotiated evidence that is attainable using deliberative methods and that may have a central role to play in the future of disinvestment policymaking. The ART evidence base, in terms of safety and effectiveness, is relatively well dotted with signposts from which to navigate a debate on these complementary qualitative, experiential matters. Not all existing health care services are as well informed, so it may be that a yet-unknown threshold of safety/effectiveness evidence is required such that productive engagement at this level is possible. Where the technical evidence is relatively robust, our results highlight that democratically informed disinvestment decision-making is feasible. In addition, it is revealed that such decisions could be potentially less controversial than often presumed [11,12,22], including for such highly ethically charged cases as ART.

## Conclusion

Deliberative processes, engaging key stakeholders including citizens, in decision making around potential disinvestment from low value health care, provides an avenue for contributing to policy making in a contentious area of health policy.

## Endnotes

^1^Through its universal health insurance program, Medicare, the Australian Government provides one of the world’s most comprehensive subsidization programs for ART procedures, for all citizens and permanent residents. Co-payments are generally required.

^2^Quality adjusted life years, a measure of disease burden.

## Competing interests

The authors declare no competing interests. The funding organisation (NHMRC) had no role in the study design, data collection, analysis and interpretation, or the writing and publication of this article.

## Authors’ contributions

JEH, AGE, ABM and JRM are Chief Investigators of The ASTUTE Health Study and as such were responsible for the initial grant writing and methodological planning. They were later joined by JS, DC, KH and AW to further plan and carry out these engagement sessions. All authors made substantial contributions to conception and design, acquisition of data, analysis and interpretation of data (with KH as principal analyst); and were involved in drafting the manuscript and revising it critically for important intellectual content. All authors read and approved the final version of the manuscript.

## Authors’ information

The ASTUTE Health Study (Assessing Services and Technology Use To Enhance Health) is a multidisciplinary research project funded by Australia’s National Health and Medical Research Council (Grant ID 565327). Authors have training in epidemiology, health technology assessment, health policy, health economics, bioethics, qualitative research, and deliberative engagement.

## Pre-publication history

The pre-publication history for this paper can be accessed here:

http://www.biomedcentral.com/1472-6963/14/204/prepub
